# Editorial: Antigen presentation in cancer immune responses

**DOI:** 10.3389/fimmu.2025.1558249

**Published:** 2025-01-27

**Authors:** Marie-Liesse Asselin-Labat, Megan K. Ruhland, Stephen T. Ferris

**Affiliations:** ^1^ Personalised Oncology Division, The Walter and Eliza Hall Institute of Medical Research, Melbourne, VIC, Australia; ^2^ Department of Medical Biology, The University of Melbourne, Melbourne, VIC, Australia; ^3^ Department of Cell, Developmental and Cancer Biology, Oregon Health & Science University, Portland, OR, United States; ^4^ Knight Cancer Institute, Oregon Health and Science University, Portland, OR, United States; ^5^ Department of Molecular Microbiology and Immunology, Saint Louis University School of Medicine, St. Louis, MO, United States

**Keywords:** dendritic cells, cancer vaccine, adaptive immunity, cancer immunology, antigen presentation, neoantigen

Antigen presentation is critical to initiate the adaptive immune response to cancer, instigating the immune response by processing tumor antigens and presenting them to T cells of the adaptive immune system. This Research Topic explores multiple facets of antigen presentation in cancer, ranging from pathways that regulate dendritic cell (DC) functions to innovative strategies for enhancing antigen presentation and developing novel vaccine designs. These articles provide new perspectives and novel approaches to harness DC-mediated antigen presentation and improve overall anti-tumor immunity.

Chen et al. set the scene with a comprehensive review of DCs, professional antigen-presenting cells essential to activate CD4^+^ and CD8^+^ T cells-mediated tumor cell killing. The review highlights the different types of DC (conventional DC: cDC1, cDC2, cDC3; monocyte-derived DC: moDC; plasmatoid DC: pDC), and addresses their known roles in the tumor microenvironment (TME). The review also provides an insightful discussion of the limitations of past and current DC therapy design and details both the needs and opportunities presented by the therapeutic strategies in development that would enable more robust exploitation of anti-tumor DC functions.

This Research Topic includes five primary research articles which together evaluate novel DC characteristics within the TME and tackle the limitations currently associated with their therapeutic use. Articles by Flynn et al. and Flores-Santibanez et al. evaluate DC properties and their regulation in the context of tumor mutations and within the TME. Flynn et al. detail DC phenotypes and function in FLT3 (Fms-like tyrosine kinase 3)-mutated acute myeloid leukemia (AML). Patients with FLT3-mutated AML, including FLT3-ITD (Internal Tandem Duplication) have a poor clinical prognosis, but little is known about the role of FLT3-ITD mutation in DCs and its impact on disease progression. By analyzing patient bone marrow and utilizing sophisticated transgenic animal models, the authors show that FLT3-ITD leads to disrupted DC homeostasis, favoring the expansion of poorly differentiated cDCs with a precursor phenotype. This alteration promoted the polarization of CD4^+^ T cells towards Th17 and Treg phenotypes, that may contribute to a tumor-supportive TME in AML.

DCs in the TME may have distinct properties from DCs in healthy organs. To address this question, Flores-Santibanez et al. evaluated the IRE1/XBP1 (inositol-requiring enzyme 1/X-Box binding Protein 1) pathway in DCs infiltrating tumours. This pathway is a cellular response maintaining the fidelity of the cellular proteome and regulates antigen cross-presentation in healthy organs. The authors showed that the regulation of immunosuppressive genes by IRE1/XBP, described in other myeloid and lymphoid cells infiltrating the TME, is not observed in tumor-infiltrating cDC1s, proposing that the pro-tumorigenic role of the IRE1/XBP is not general but rather cell-type and context-dependent.

Studies on increasing cDC1 activation and antigen presentation are critical to improve the efficacy of cancer vaccines. cDC1s express the chemokine receptor XCR1, making it an interesting target to direct therapy specifically to these cells. Given that type I interferon (IFN) is a potent activator of innate immune cells, targeting type I IFN to cDC1s exclusively would activate cDC1s but avoid the toxic effects of systemic pro-inflammatory type I IFN. To this end, Noe et al. engineered an XCR1 antibody fused to IFN-mutein to potentiate IFN activity in cDC1s. While the antibody activated cDC1s, repeated dosing resulted in strong anti-drug antibodies, with a loss of drug activity. Their data demonstrate that caution needs to be taken when targeting immunostimulatory agents to cDC1s due to their effectiveness in inducing an anti-drug antibody response.

Innovative research by Morisaki et al. demonstrated the potential of hybrid neoantigen peptide-pulsed dendritic cell (DC) vaccines in stimulating robust anti-tumor immune responses. These peptides, designed to be presented by MHC-I and MHC-II, showed remarkable efficacy in activating both CD4^+^ and CD8^+^ T cells through direct antigen presentation and cross-presentation, respectively. *Ex vivo* analysis of patient samples revealed increased T cell responses against multiple class I and class II neoepitopes following vaccination, highlighting the vaccine’s ability to induce a diverse and potent immune response.

The article by Azzi et al. describes a non-classical approach to activating the anti-tumor response by increasing MHC-II expression in tumor cells in a model of oral squamous cell carcinoma, forcing tumor cells to act as antigen presenting cells (APCs). This study is supported by previous literature showing the expression of MHC-II in tumor cells is associated with better clinical outcomes (1–3). Azzi et al. suggest that by overexpressing CIITA, the master regulator of MHC-II expression, tumor cells can function as APCs to support tumor-specific immune responses. Further studies *in vivo*, in which DCs are depleted, will be necessary to determine whether tumor-induced MHC-II expression is sufficient to trigger CD4^+^ and CD8^+^ T cells-mediated anti-tumor immunity.

Cancer vaccines are showing promises as the next generation of cancer immunotherapies. The success of these therapies will rely on identifying the right neoantigens but also ensuring effective antigen presentation for T cell activation. Strategies to enhance antigen presentation and T cell priming within the immunosuppressive TME will be essential for improving therapeutic outcomes. As highlighted by Chen et al., more research is needed to better understand tumor antigen presentation and boost anti-tumor immunity. Future research into improving antigen presentation and anti-tumor immune responses will likely focus on addressing outstanding questions that have emerged from the recent studies in this Research Topic: (1) optimizing antigen delivery methods to improve DC targeting and activation without stimulating an anti-drug antibody response, (2) elucidating the role of non-classical antigen presentation pathways in anti-tumor immunity, and (3) developing combination therapies that synergize with antigen presentation-based approaches. These advancements may lead to more effective and personalized cancer immunotherapies, potentially improving outcomes for patients across various cancer types.
